# Mortality and mental health funding—do the dollars add up? Eating disorder research funding in Australia from 2009 to 2021: a portfolio analysis

**DOI:** 10.1016/j.lanwpc.2023.100786

**Published:** 2023-05-16

**Authors:** E. Bryant, N. Koemel, J.A. Martenstyn, P. Marks, I. Hickie, S. Maguire

**Affiliations:** a InsideOut Institute, Charles Perkins Centre, Faculty of Medicine and Health, University of Sydney and Sydney Local Health District, Australia; b The Boden Initiative, Charles Perkins Centre, Faculty of Medicine and Health, University of Sydney, Australia; c School of Psychology, Faculty of Science, University of Sydney, Australia; d Brain and Mind Centre, Faculty of Medicine and Health, University of Sydney, Australia

**Keywords:** Portfolio analysis, Medical research funding, Eating disorders, Mental health, Health policy, Disease burden, Health economics, Anorexia nervosa, Bulimia nervosa, Binge eating disorder, Mental health funding, Research funding, Economic analysis, Financial

## Abstract

**Background:**

Eating Disorders (EDs) are among the deadliest of the mental disorders and carry a sizeable public health burden, however their research and treatment is consistently underfunded, contributing to protracted illness and ongoing paucity of treatment innovation.

**Methods:**

We compare absolute levels and growth rates of Australian mental health research funding by illness group for the years 2009–2021, with a specific focus on eating disorders analysed at the portfolio level.

**Findings:**

Actual and adjusted data obtained from Australia's three national medical research funding bodies (NHMRC, ARC and MRFF) shows eating disorders receive a disproportionately low allocation of mental health research funding despite having amongst the highest mortality rates. Forty-one category one research grants totalling $AUD28.1 million were funded for eating disorders over the period. When adjusted for inflation, this equates to $2.05 per affected individual, compared with $19.56 for depression, $32.11 for autism, and $176.19 for schizophrenia. Half of all research funded for eating disorders was ‘basic’ research (e.g., illness underpinning), with little investment in the development of innovative treatment models, novel therapeutics or translation, well reflected by recovery rates of less than 50% in individuals with Anorexia Nervosa.

**Interpretation:**

Significant discrepancy remains between research funding dollars and disease burden associated with the mental health disorders. The extent to which eating disorders are underfunded may in part be attributable to inaccuracies in epidemiological and burden of disease data.

**Funding:**

This work was in-part funded by the 10.13039/501100003921Australian Government Department of Health and the National Eating Disorder Research & Translation Strategy. The funder was not directly involved in informing the development of the current study.

**Research in context**

**Evidence before this study**

Research in Canada, the US and the UK shows a disproportionately low allocation of research funding devoted to eating disorders despite their high prevalence, high mortality and significant personal and public health burden. No studies have examined funding at the portfolio level, been conducted in Australia, or comprehensively explored the extent to which inaccuracies in epidemiological and burden of disease data lead to investment shortcomings.

**Added value of this study**

This study provides a detailed picture of investment allocations and type of research funded for eating disorders in Australia. It explores the structural exclusion and data shortcomings that contribute to underfunding and the impact this has on treatment innovation and chronic illness, providing suggestions for systemic, whole-of government change.

**Implications of all the available evidence**

A systemic whole-of-government approach is needed to address inadequate investment in mental illness more broadly and particularly for illness groups such as eating disorders where complex structural limitations of disease burden and mortality reporting exist. The establishment of dedicated funding initiatives, a focus on funding novel therapeutics and rapid standardisation of data collection practices across healthcare services are urgently needed to overcome existing epistemic barriers to investment and prevent serious personal and public burden.

## Introduction

Despite increased awareness and advocacy prompted by escalating primary care presentations^1^, ^2^, ^3^, ^4^ and persistent rising health system costs^5^ there remains continued failure to adequately fund mental health research and service provision. A significant incongruence exists between global burden of disease and the proportion of health budgets devoted to preventing and treating mental disorders.^6^, ^7^, ^8^, ^9^, ^10^ In Australia, mental ill-health causes the third highest all-age disability burden, and notably, the highest for those aged 15–44.^11^ In the most recent national mental health survey, 39.6% of young people aged 16–24 met survey criteria for a mental disorder in the last 12 months.^12^ Mental ill-health is responsible for up to 22% of total disease burden in established market economies^6^ and is the leading cause of non-fatal health loss globally.^13^ An identified National Health Priority Area and both an independent and comorbid risk factor for physical disease of almost every major organ system, mental health receives less than 10% of total public medical research funding.^8^^,^^14^ Meanwhile, the health system cost of mental disorders doubles every seven years.^15^

In 2021, 18 disease-specific areas were funded a total of over 1.35 billion by the National Health and Medical Research Council (NHMRC), whilst broad mental health received approximately 7.5% of this funding at $102.3 million (Table 1). Additionally, mental health research in Australia, and internationally, is poorly supported by philanthropic, foundation private sources and industry-directed funding as well as being limited by scarce large-scale publicly funded specialist health services, where clinical research is typically enabled.

**Table 1:** NHMRC expenditure for disease, research and health areas 2013–2021 ($AUD millions).

Disease, research and health areas^a^	2013	2014	2015	2016	2017	2018	2019	2020	2021
Balance, eye and hearing diseases	25.6	27.5	28.9	26.3	22.2	21.8	21.3	22.3	22.7
Blood diseases	23.5	26.3	24.6	23.8	24.8	20.0	23.3	24.0	21.9
Cancer	179.2	188.3	191.4	170.6	175.8	178.9	181.6	170.2	153.7
Cardiovascular disease	117.1	129.4	130.0	114.9	111.4	105.3	112.6	107.6	102.5
Congenital and genetic diseases	93.4	106.7	117.9	112.6	102.1	104.3	112.6	104	90.1
Endocrine, metabolic and nutritional diseases	129.9	137.5	139.9	120.2	111.5	104.3	117.5	115.2	109.5
Environmental and occupational health	16.9	20.4	18.2	17.8	15.2	17.2	16.9	19.1	19.3
Gastrointestinal diseases	39.1	39.3	41.7	35.7	34.9	37.5	42.2	44.7	44.3
Genitourinary diseases	38.2	41.6	41.0	38.6	39.0	40.0	41.4	42.1	35.0
Immunological diseases^b^	84.2	94.9	99.2	85.1	71.1	79.5	81.0	79.1	70.9
Infectious diseases	143.8	158.2	167.4	148.7	148.7	159.4	159.4	165.3	161.6
Injury	45.4	58.4	61.5	45.8	44.2	49.9	49.9	49.8	46.6
Mental health^c^	85.1	95.9	100.0	91.1	93.4	104.9	110.2	103.8	102.3
Musculoskeletal diseases	52.5	58.5	61.9	49.3	49.2	51.9	49.7	46.1	41.7
Neurological diseases	167.0	196.2	214	198.8	190.1	204.3	214.6	210.2	196.1
Orofacial diseases	7.0	7.2	6.8	3.9	3.4	3.3	3.4	2.3	2.6
Reproductive health	72.6	77.8	83.6	66.6	62.8	65.2	69.5	71.1	68.3
Respiratory diseases	56.4	63.5	68.3	51.2	46.4	50.8	55.8	56.6	57.7
Skin diseases	15.4	20.0	16.6	12.8	9.5	10.9	11.2	10.4	11.1

a These disease, health and research topics are based on the International Classification of Disease (ICD) produced by the World Health Organization.

b The figures in the table above for Immunological Diseases have been modified to exclude immunological research specifically related to cancer. These figures relate to research relevant to allergy, autoimmune diseases, and immunodeficiency.

c Includes research into addiction.

Some groups within mental health face particularly stark funding disparity relative to disease burden and mortality, including Trauma, Personality and Eating Disorders (EDs).^10^ For EDs, stigma and ignorance of illness complexity are exacerbated by their frequent misrepresentation in public discourse.^16^, ^17^, ^18^, ^19^ A lack of robust epidemiological data, absence from national surveys informing policy and funding^20^ and significant methodological limitations in studies of disease burden may also be major factors in the under resourcing of these illness groups.

EDs (including Anorexia Nervosa, Bulimia Nervosa, Binge Eating Disorder, Avoidant Restrictive Food Intake Disorder, Other Specified Feeding or Eating Disorder and Unspecified Feeding or Eating Disorder) are complex psychiatric illnesses associated with numerous medical sequalae.^20^, ^21^, ^22^ They have a population prevalence of approximately 4–5%,^23^^,^^24^ consistently poor outcomes^25^, ^26^, ^27^, ^28^, ^29^ and alarmingly high mortality rates^22^^,^^30^^,^^31^ (Table 2), but have been conspicuously absent from major research and public health initiatives, and their research and treatment consistently underfunded. Among the most disabling of the mental disorders (anorexia nervosa is associated with a higher mortality rate, carer and healthcare burden than both schizophrenia and depression^32^, ^33^, ^34^, ^35^), the ED research dollar spend worldwide is not commensurate. In the US, ED research receives US$0.73 per affected individual, while autism research receives US$58.65 per affected individual, and schizophrenia research US$86.97 per affected individual.^36^ In 2016, it was estimated that around 20 million people in the European Union (EU) had an ED at a fiscal cost of €1 trillion per year (€249 billion in financial costs plus approximately €763 billion in disease burden). That same year, just 0.4% of the UK's mental health research expenditure went to EDs, compared with 7.2% for depression and 4.9% for psychosis.^37^ In 2017/18, Canada spent an average of $CAD0.59 on ED research per affected individual but $CAD24,017 on treating them—1.79 times the healthcare cost of an individual with schizophrenia^38^ (Stone et al.). In Australia, the combined social and economic cost of EDs has been estimated to be $AUD69.7 billion, with a $AUD100 million annual burden on the healthcare system,^24^ likely increased since the emergence of the COVID-19 pandemic has seen burgeoning ED presentations in primary and tertiary care settings.^39^^,^^40^ Incidence of other specified eating disorders and eating disorders in younger age groups continues to increase.^41^, ^42^, ^43^

**Table 2:** Standardised mortality ratio (SMR) for major mental disorders reported by large-scale epidemiological studies or meta-analyses with up to 30 years follow-up.^30^^,^^31^^,^^44^, ^45^, ^46^, ^47^, ^48^, ^49^, ^50^, ^51^, ^52^, ^53^, ^54^, ^55^, ^56^, ^57^, ^58^

Eating disorders					Schizophrenia	Depression	Anxiety disorders
	All ED	AN	BN	OSFED			
Zilber et al., 1989					6.33	8.53	
Emborg, 1999	6.69	22.03					
Löwe et al., 2001		9.80					
Birmingham et al., 2005		10.50					
Chang et al., 2010						1.29	
Arecelus et al., 2011		5.86		3.30			
Rosling et al., 2011	10.00	11.70					
Khan et al., 2013					1.32–2.83	1.59–2.02	0.90–1.88
Cuijpers., 2014						1.64	
Keshaviah et al., 2014		5.17					
Olfson et al., 2015					3.70		
Walker et al., 2015					2.54	1.71	1.43
Fichter & Quadflieg 2016			1.49	2.39			
Pratt et al., 2016						1.6	
Heiberg et al., 2018					4.9		
Tanskanen et al., 2018					2.7		
Lomholt et al.m 2019					4.58		

AN = Anorexia Nervosa; BN = Bulimia Nervosa; ED = Eating Disorders; OSFED = Other Specified Feeding or Eating Disorder.

The standardised mortality ratio (SMR) for anorexia nervosa (AN) is repeatedly found in large-scale epidemiological studies to be higher than all of the other major mental disorders^59^ (Table 2). Reported SMRs range between 5.17 and 22.03.^30^^,^^31^^,^^44^, ^45^, ^46^, ^47^, ^48^ For depression, reported SMR ranges between 1.29 and 8.53^49^, ^50^, ^51^, ^52^, ^53^, ^54^; for schizophrenia 1.32–6.33,^51^^,^^53^, ^54^, ^55^, ^56^, ^57^, ^58^ and for anxiety disorders 0.90–1.43.^51^^,^^53^ AN has a crude mortality rate of up to 22%.^59^

Despite this, structural (or system-level) exclusion of eating disorders has been widely reported.^20^^,^^22^^,^^60^^,^^61^ EDs were not included in any Australian national mental health plans prior to 2017; they have almost always been omitted from national surveys of mental wellbeing and remain excluded at the time of writing. Additionally, no ED healthcare data is nationally centralised or mandatorily collected, despite the illness group being the only mental disorder to have been given its own Medicare item numbers, quadrupling the number of rebated psychological sessions available for moderate to severe cases.^62^ This means most prevalence data is estimated or derived from small population or cohort studies, resulting in large uncertainty intervals and underpowered burden estimates and there is little data on who is presenting to ED services, what treatment they receive and whether or not that treatment is effective.^63^

Notwithstanding the limited funding available, Australian researchers have made significant contributions to the knowledge base relating to sociocultural risk factors for body dissatisfaction and eating disorders; the development of body image attitudes in children; evaluation of prevention and early intervention strategies, and the understanding of the role of media and social media, among others.^64^ ED publications by Australians have been increasing since 1990 in line with international rates, however, a significant amount of unfunded research is occurring. In 2019, just over 18% of all ED publications were identified as being from funded ED research.^64^

### Medical research funding in Australia

Publicly-funded medical research in Australia has historically been largely provided by two government bodies—the NHMRC and the Australian Research Council (ARC). Together, these are known as ‘category one’ funding initiatives. They grant up to $1.5 billion a year for new projects and $5 billion to existing projects in the basic sciences, clinical medicine and science, health services and public health research fields. This research aims to inform evidence-based practice, reduce health risk factors and inequities, and contribute to the understanding of biological processes that underpin diagnostic and therapeutic innovation. A third category one government initiative—the substantial $20 billion Medical Research Future Fund (MRFF)—was announced in the 2014–2015 federal budget as an ongoing commitment to medical research that drives innovation, health system change and economic growth.^65^^,^^66^ The MRFF is typically allocated via targeted funding initiatives. These initiatives are guided by expert advisory panels such as the Genomics Health Futures Mission, the Australian Brain Cancer Missions Strategic Advisory and the Million Minds Mental Health Research Mission.^66^ Smaller granting bodies in Australia include the Biomedical Translation Fund, Australian Rotary Health, public and private research organisations such as the Commonwealth Scientific and Industrial Research Organization (CSIRO) and Cancer Australia; schemes including indirect block grants to universities and Research Development Tax Incentives; and finally philanthropic charities and Not-For-Profits (NFPs).

When identifying priority areas for medical research, funders must balance public health need, disease burden and severity with scope for impact and translation.^67^^,^^68^ Since the late 1980s, health policy documents have been developed by the state and federal governments to guide allocation of financial resources for medical research.^69^, ^70^, ^71^ These guidelines broadly identify burden of disease; ‘areas of unmet need’; and potential health and economic benefit as primary considerations in the allocation of resources.^67^^,^^68^^,^^71^, ^72^, ^73^

### The role of burden of disease in medical research funding

**Fig. 1:**
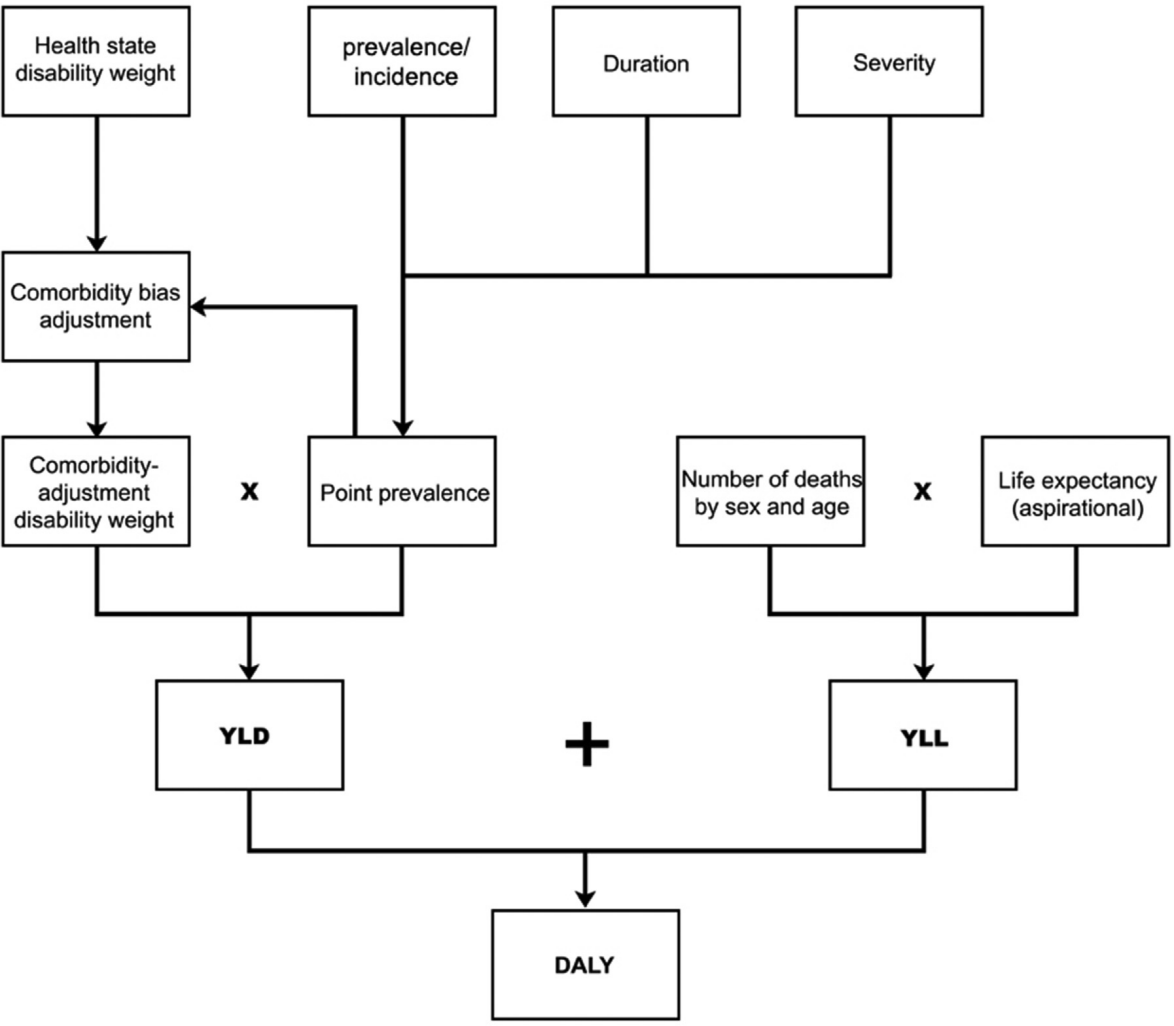
Overview of disability-adjusted life year estimation process, Australian Burden of Disease Study 2018 (AIHW, 2022).^74^

Burden of Disease (BoD) estimates are a primary consideration when it comes to medical research funding. These are population health summary measures based on data from death and disease registries, health facilities and population surveys. Mortality alone does not give a complete picture of the burden of disease. BoD is a summary metric of disability-adjusted life years (DALY)—a time-based measure that combines years of life lost due to premature mortality (YLLs) and years of life lost due to time lived in states of less than full health, or years of healthy life lost due to disability (YLDs) (see Fig. 1). One DALY represents the loss of the equivalent of one year of full health. If a disease has a high number of DALY, it is considered to have a high burden on the population.

BoD estimates are not without methodological limitation.^75^, ^76^, ^77^ Non-fatal disease burden (YLD) is commonly reported with significant uncertainty intervals due to data shortcomings and variations in case definition and measurement of important aspects of illness.^78^ Measurement of YLD involves the algorithmic quantification and summation of point prevalence rates, severity indications and disability weightings.^74^ Both the Australian and Global Burden of Disease studies use methodology derived from a 2010 multi-national study (updated in 2013) which models pairwise comparisons rated by members of the general public to assign disability weightings on a scale from 0 (no health loss) to 1 (total health loss).^75^^,^^78^, ^79^, ^80^ This weighting is multiplied by the prevalence of the sequela without age weighting or discounting to arrive at a YLD.^81^ This methodology is contentious for several reasons including the brevity of the lay descriptions which cannot adequately capture detailed symptoms and impacts of a disorder (see for example Table 3 the descriptions of anorexia and bulimia nervosa^78^), the infrequency with which the validity of these health state descriptions have been evaluated or reported, and lack of expertise among the general population about specific health states.^82^ Making equivocal assumptions about how harms of differing magnitudes should be aggregated may be particularly problematic for mental disorders, where the impacts of illness are frequently misunderstood and can affect psychosocial functioning in a way that cannot be easily quantified.^83^ YLDs therefore are almost certainly underestimated for mental illness.

**Table 3:** Illness descriptions of AN and BN for the purposes of assigning disability weighting, Global Burden of Disease Study 2013 collaborators.^78^

1.1. Anorexia nervosa	Feels an overwhelming need to starve and exercises excessively to lose weight. The person is very thin, weak and anxious.
1.2. Bulimia Nervosa	Has uncontrolled overeating followed by guilt, starving and vomiting to lose weight.

### Objective

To inform the Australian Eating Disorder Research and Translation Strategy 2021–2031^64^ and the consideration of its viability in the next decades, InsideOut Institute conducted a thorough analysis of ED research funding for calendar years 2009–2020. This analysis was updated for the current study to include 2021. It is the first to thoroughly analyse ED research funding in Australia at the portfolio level and to consider the impact of data shortcomings on current measurements of disease burden.

### Role of the funding source

This work was in-part funded by the Australian Government Department of Health and the National Eating Disorder Research and Translation Strategy. The funder was not directly involved in informing the development of the current study.

## Methods

Descriptive analyses of awarded Category One medical research funds were conducted for the years 2009–2021 using data obtained from the NHMRC, and through independent keyword analysis of publicly available MRFF and ARC datasets, in order to describe and document trends.

### Funding by illness group

NHMRC data analysts provided total grant funding by illness group which included committed and expended funds per calendar year. MRFF and ARC datasets were filtered by field of research for biomedical, health, and related medical sciences and then searched using the following search terms and Boolean operators (eating disorder + anorexia + bulimia + binge eating disorder + ARFID + UFED + disordered eating + body image) OR (depression + depressed + depressive + mood + affective + dysthymia) OR (anxiety + anxious + generalised anxiety disorder + phobia + PTSD + post-traumatic stress + trauma + obsessive compulsive + agoraphobia + social + panic) OR (autism + ASD + spectrum + Asperger's + pervasive developmental + neurodivergent) OR (schizophrenia + psychosis + psychotic + schizoaffective + affective + delusion + mania). All grant descriptions and summaries were appraised by two researchers (EB & JM) and included or excluded according to the following criteria.

#### Inclusion/exclusion of grants

Awarded grants were included within their relevant category for that year if:

a) The aim was directly related to the understanding, risk identification, prevention or treatment of the mental disorder (e.g., the evaluation of an eating disorder prevention or treatment program); OR
b) The aim was indirectly related to the understanding, risk identification, prevention or treatment of the mental disorder (e.g., the development of a healthy body image program for adolescents).

Grants were excluded if there was no direct or obvious indirect connection to the mental disorder (for example, biomedical research grants which included the keyword ‘anorexia’ referring to loss of appetite in the context of cancer or other pathophysiology).

To calculate research spend per affected individual, total awarded funding for the three major awarding schemes (NHMRC, ARC and MRFF) was cross-tabulated by illness group and official point prevalence rates reported by the Australian Bureau of Statistics (ABS) where available (Major Depressive Disorder—4.6%, and Anxiety Disorders—16.8%^12^) and the Department of Health for other illness groups (Autism—0.7%^84^ and Schizophrenia—0.31%^85^). Where such data does not exist (EDs), a point prevalence rate of approximately 4.5% was ascertained from large epidemiological studies and reports.^23^^,^^24^ As costs measured in different years are not directly comparable, funding was adjusted to calendar year 2021-equivalent using an a priori method consistent with previous research (Williams, Pearce & Smith, 2021; Lystad et al., 2020; Handelsman 2012)^86^, ^87^, ^88^ using the Reserve Bank of Australia Inflation Calculator available from the Australian Government (https://www.rba.gov.au/calculator/).^89^^,^^90^ One-way ANOVA with group comparisons was performed to assess for significant total funding differences across illness group. Linear regression was used to assess for any statistically significant changes over time in the proportion of research funding provided to mental disorders overall and repeated with sub-group analyses for each illness group.^70^ A correlation coefficient (r) of 1 would indicate a 100% positive relationship between time and research funding allocated and −1 would indicate a 100% negative relationship between time and research funding allocated. All statistical analyses were undertaken with SPSS (Version 28; IBM Corporation; Armonk, NY). Statistical significance was declared at p < 0.05.

### Eating disorder portfolio analysis

Retrospective portfolio analysis was conducted on those grants awarded to ED research. All identified ED grants were coded by two researchers for research topic using the UK Health Research Classification System (HRCS) Research Activity Code^91^; mapped against the Top 10 Research Priorities for Eating Disorders in Australia^92^ (see Table 4), and assessed for inter-rater reliability using percentage agreement.

**Table 4:** HRCS research activity codes and corresponding eating disorder research priorities.

HRCS RAC9^91^	Definition	Top 10 research & translation priorities^92^
1. Underpinning	Research that underpins investigations into the cause, development, detection, treatment and management of diseases, conditions and ill health	Risk & protective factors
2. Aetiology	Identification of determinants that are involved in the cause, risk or development of disease, conditions and ill health	Risk & protective factors Early identification
3. Prevention & promotion of wellbeing	Research aimed at the primary prevention of disease, conditions or ill health, or promotion of well-being	Prevention Stigma & Health promotion
4. Detection, screening and diagnosis	Discovery, development and evaluation of diagnostic, prognostic and predictive markers and technologies	Early identification Early intervention
5. Development of treatments and therapeutic interventions	Discovery and development of therapeutic interventions and testing in model systems and preclinical settings	Individualised medicine
6. Evaluation of treatments and therapeutic interventions	Testing and evaluation of therapeutic interventions in clinical, community or applied settings	Treatment outcomes
7. Management of disease	Research into individual care needs and management of disease, conditions or ill health	Support families
8. Health and social care services	Research into the provision and delivery of health and social care services, health policy and studies of research design, measurements and methodologies	Equity of access Do no harm

#### Coding and analyses

Each grant included in this analysis was independently coded by two authors (EB and JM) in April 2022. Inter-rater reliability was high with 80% agreement at first review across all grants. A second review involved discussion and re-appraisal of research activity codes for grants there was disagreement on at first review. Relative proportions of eating disorder research funding allocated to each HRCS Research Activity/Top 10 Research priorities were analysed. These were based on cash investments only; in-kind contributions to were not included. Descriptive analyses are presented.

## Results

### Total funding by illness group

**Fig. 2:**
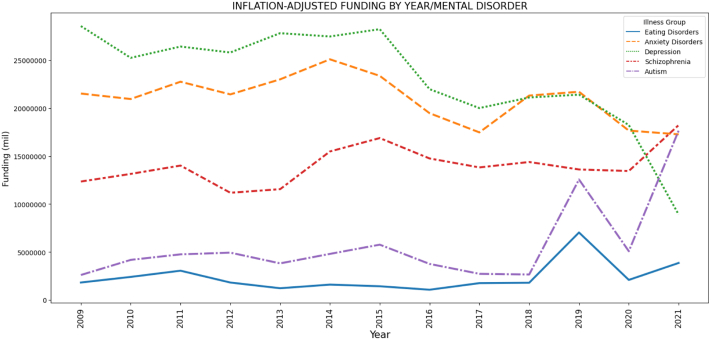
NHMRC (incl. MRFF) and ARC funding per year, by mental disorder, 2009–2021, adjusted for inflation (2021 equivalent, Australian dollars (AUD)).

Between 2009 and 2021, 41 research grants totalling $28.12 million were awarded for ED research in Australia. Of these 41 grants, 30 were funded by the NHMRC (for a total investment of $19.70 million), 3 by the MRFF (total investment = $6.19 million) and 8 by the ARC (total investment = $2.23 million). By comparison, research grants totalling $266.45 million, $243.69 million, $69.43 million, and $164.71 million were awarded for Depression, Anxiety Disorders, Autism and Schizophrenia respectively (Fig. 2). Group comparisons showed significant differences in funding received by illness group (p < 0.05), with a total inflation-adjusted spend equating to $2.05 per affected individual for eating disorders; $4.86 per affected individual for anxiety disorders; $19.56 per affected individual for depression; $32.11 per affected individual for autism and $176.19 per affected individual for schizophrenia.

Linear regression analyses showed research investment in each major mental illness group except EDs (p = 0.11) changed significantly over time (Table 5). Funding awarded to depression (*r* = −0.810) and anxiety disorders (*r* = −0.559) was dramatically reduced; for schizophrenia (*r* = 0.526) and autism (*r* = 0.567) was moderately increased; and for EDs stayed the same except for a spike in 2019 (*r* = 0.364).

**Table 5:** NHMRC (incl. MRFF) and ARC funding per year (AUD), by mental disorder, 2009–2021 (millions), adjusted for inflation (2021 equivalent).

Illness group	Total funding (unadjust.) ($)	Average funding/yr ($)	Inflation-adjusted funding/yr ($)	Point prevalence^b^	Per individual ($)	Change over time^c^ (r)	Sig.^a^
Schizophrenia	164,715,246.60	12,670,403.58	14,064,003.66	0.31%	176.19	0.526	0.03^a^
Autism spectrum disorders	69,433,719.00	5,341,055.31	5,787,029.30	0.7%	32.11	0.567	0.02^a^
Depression	266,448,196.00	20,496,015.08	23,173,872.78	4.6%	19.56	−0.810	<0.001^a^
Anxiety disorders	243,686,521.00	18,745,117.00	21,004,278.74	16.8%	4.96	−0.559	0.02^a^
Eating disorders	28,118,796.20	2,162,984.32	2,374,780.92	4.5%	2.05	0.364	0.11

a Two-tailed.

b See methods for citations.

c Correlation coefficient.

### Portfolio analysis

The majority of grants awarded to ED research were distributed to ‘Underpinning’ (*n* = 16), or the ‘research that underpins investigations into the cause, development, detection, treatment and management’ of eating disorders (Table 6). Much of this research is laboratory or ‘bench’ based including investigations into molecular, cellular, and physiological structures and function; biological pathways and processes; social and cultural beliefs; and perception, cognition and learning processes. ‘Evaluation of treatments and therapeutic interventions’ (*n* = 7) and ‘Prevention, promotion of wellbeing’ (*n* = 7) were the second most awarded categories. This includes clinical and applied application and evaluation of psychological, pharmacological, physical or complementary interventions; and research aimed at primary prevention interventions to modify behaviours (including risk behaviours), improve public health policy and education or promote wellbeing. The third most common type of research to be funded was aetiological research (*n* = 5), including investigations into biological, endogenous, environmental, and psychosocial contributors to eating disorders. Research areas with the lowest number of awarded grants included ‘Detection, screening & diagnosis’ (*n* = 2), ‘Health and social care services’ (*n* = 2), and ‘Management of disease’, which was not funded at all.

**Table 6:** Total number of awarded grants by HRCS research activity code 2009–2021.

HRCS Research activity code (RAC)	NHMRC	MRFF	ARC	Total No. Awarded	Average investment/project ($AUD)	Median investment/project ($AUD)
1. Underpinning	16	0	0	16	707,164.23	782,185.00
2. Aetiology	3	0	2	5	538,498.72	204,186.50
3. Prevention and promotion of wellbeing	3	0	4	7	455,332.06	360,000.00
4. Detection, screening & diagnosis	1	0	1	2	350,167.76	97,874.00
5. Development of treatments and therapeutic intervention	2	1	1	4	829,497.23	896,518.00
6. Evaluation of treatments and therapeutic interventions	6	1	0	7	612,594.96	1,175,522.00
7. Management of disease	0	0	0	0	0.00	0.00
8. Health and social care services	1	1	0	2	2,038,960.70	3,670,400.00
9.Not ED	−2	0	0	−2	N/A	
**TOTAL**	**30**	**3**	**8**	**41**	**Total average.** **691,526.96**	**Average median.** **898,335.71**

HRCS = Health Research Classification System; ED = Eating Disorder.

Different types of research require inherently different levels of investment due to variations in methodology and resourcing costs. Average investment per research project was calculated for each HRCS activity code. Investment per project was lowest for ‘Detection, screening and diagnosis’ (mean $350,168, median $97,874), and highest for ‘Health and social care services’ (mean $2.04 mil, median $3,670,400)—the latter was due to one large MRFF investment ($3.6 million) awarded to the University of Sydney to model population prevalence and health system usage data for eating disorders. Average investment per project was also higher for research into the ‘Development of treatments and therapeutic interventions’ (median $896,518; mean $829,497), and ‘Underpinning’ (median $782,185; mean $707,164). The total average investment in any eating disorder research project was $691,527 (median $898,336).

**Fig. 3:**
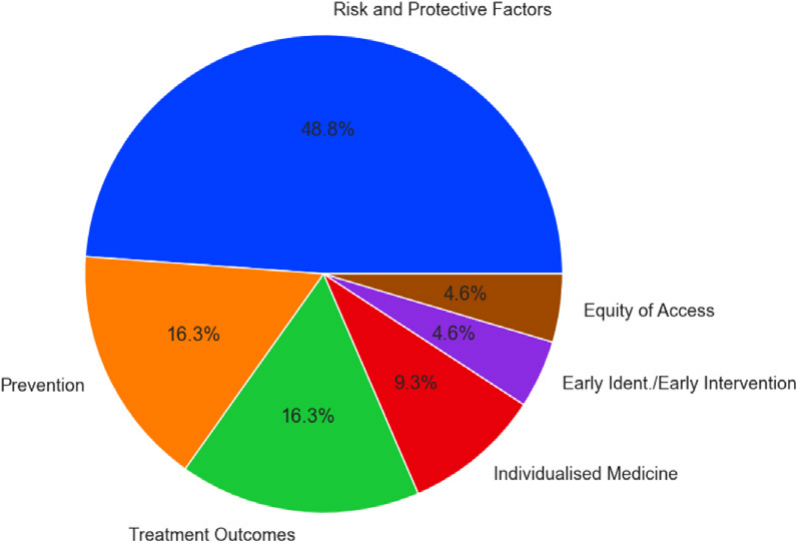
Awarded grants by Top 10 research priority area 2009–2021 (%).

When stratified against the Top 10 research priority areas for eating disorders, over 65% of all research funded in the last decade and a half has been provided for the study of premorbid factors—i.e., Risk Factors and Prevention. Less than 17% of funding has gone towards research into current treatment outcomes, <10% to projects on individualised medicine and <5% each for the investigation of early identification/intervention and equity of access (Fig. 3).

## Discussion

Significant discrepancy remains between research funding dollars allocated to illness groups relative to their BoD estimates, and indeed their actual disease burden. This is particularly stark in the mental illnesses, and eating disorders are no exception. Among the mental illnesses, research dollar allocation is not commensurate with current BoD estimates. For EDs, while the allocation may more closely align with official BoD estimates, there is a case for significant miscalculation of BoD and therefore marked underestimation of actual burden. Official BoD estimates in EDs are limited by several factors including incomplete healthcare statistics,^93^ misattributed reporting of causes of death^94^ and an almost certain underestimation of non-fatal disability^7^^,^^77^ which involves an oversimplified, high-level description of the two least common disorders to estimate disability weighting.

Several factors may be related to underestimates of official mortality and BoD in EDs. Mental disorders do not appear in large numbers in cause of death statistics anywhere.^93^ At a local (e.g., death certificate) and systemic level (e.g., OECD, WHO definition) cause of death is defined as “the disease or injury that initiated the train of morbid events leading directly to death, or the circumstances of the accident or violence that produced the injury”.^95^ This means primary diagnosis of a mental disorder is frequently subsumed under other cause of death classifications including suicide/depressive illness; chronic organ failure; malnutrition or myocardial infarction.^93^^,^^94^ This has significant implications for the under-reporting of deaths from EDs.^94^ Similarly, due to their substantial comorbidity, ED cases are frequently absorbed into other mental disorders within global GBD estimates, which are adjusted for comorbidity so that double or triple counting does not occur.^96^^,^^97^

The World Health Organization's Global Burden of Disease Study 2019 reported 95% uncertainty ranges (all ages, both sexes) for all global burden of disease estimates, calculated as 0.5∗ (upper bound –lower bound)/median value for each cause category. Uncertainty was ± 66.6% for EDs.^96^ Santomauro et al. (2021) explored these estimates of ED prevalence and disability, noting that only AN and BN are included in this study, and not the more prevalent EDs of Binge Eating Disorder (BED) and Other Specified Feeding or Eating Disorders (OSFED).^98^^,^^99^

By combining GBD database estimates with the results of 54 ED epidemiological studies, they estimated that the GBD study missed 41.9 million cases of EDs (95% uncertainty interval [UI] 27.9–59.0). When adding BED and OSFED to the estimates, the global disability adjusted life years associated with EDs almost doubled, to 6.6 million.^98^ The AIHW Australian Burden of Disease Study 2018 also includes only AN and BN in its estimates,^74^ suggesting both local and international estimates are compromised. As such, inaccuracies in the measurement of overall disease burden used to allocate research resources and funding likely plays a significant role in the low investment in EDs to date.

Further the BoD metric in health research funding in general is used inconsistently; while broad allocation of funds to priority health areas with high BoD may occur at the national and funding body level, there is evidence of a disconnect at the level of allocation of individual grants, where BoD may not be taken into consideration. A recent review of the selection criteria of all health research grant schemes listed on the Australian Competitive Grants Register found that, despite the suggested importance of BoD in funding allocation, at the funding criteria level, disease burden is often not part of the scoring matrix.^100^ Nationally the dominant criteria used to determine funding were team quality and capability (94%), research plan clarity (94%), scientific quality (92%) and research impact (92%). Disease burden was considered in only 8% of schemes.

The current analysis found that in Australia, EDs receive 1% of the research funding of schizophrenia (per affected individual), despite AN having higher mortality, carer and disability burden^32^^,^^33^; and less than 10% of the research funding of depression per affected individual. EDs was the only major mental illness group included in the analysis that saw no significant change in research investment dollars over the thirteen-year period. Notably, despite increased attention to mental health concerns and numerous calls to action, total relative investment in depression and anxiety research has dropped dramatically in the last decade. Moderate increases were observed in funding for schizophrenia and autism.

Medical research aims to improve health through innovation: that is, to advance both understanding of illness phenomena and development of treatment options for priority-driven research translation and impact that improves the health and quality of life for all individuals.^101^ Without sufficient investment, innovation within existing and emerging areas of health need is severely limited. Consistently poor investment in ED research in Australia to date means we have primarily conducted ‘basic’ research (underpinning, aetiology and prevention), with a very limited focus on translational (detection/screening, development of treatments) or applied (management of disease, health and support services) research.^10^ With most funding allocated to the prevention and understanding of what causes eating disorders, investment into understanding what happens once an individual is diagnosed is even lower than the overall dollar spend suggests. It is also worth noting that recent systematic reviews suggest limited discernible impact of prevention efforts on development of illness,^102^ highlighting further the importance of research investment for new or more effective clinical treatments. Just four projects in 13 years have been funded to develop new treatments, despite a paucity of efficacious treatments, particularly for AN, which has recovery rates in the region of just 40–50%.^27^^,^^28^^,^^47^^,^^103^

There are some limitations to this analysis. The multidisciplinary nature of research can make it difficult to accurately delineate different types of research in the health and medical field when searching datasets; equally, multi-method, multi-objective studies make the assigning of research activity code somewhat subjective or a trade-off between competing categories. Moreover, in-kind contributions, independent providers (such as Australian Rotary Health) and philanthropy were not included in the analysis. Due to lack of nationally consistent data, point prevalence rates for each of the mental disorders were reported from different years. We aimed to obtain the most accurate recent statistics for these purposes. It was not possible to code disorder-specific funding allocations within funding allocated to “eating disorders” as an umbrella group, nor would it reflect the broad categorisation of the other mental disorders captured in the analysis. Finally, lack of power due to the small number of awarded grants to eating disorder research may have impacted the analysis.

### Conclusions

Equitable, priority-driven allocation of medical research funding is essential to drive improvements in clinical care and the health of all Australians. Eating disorders are curable, however historically low investment in new clinical services and linked clinical and translational research initiatives has contributed to lack of innovation in effective treatment development and delivery, with serious ongoing implications including preventable chronic illness, high death rates and significant health system costs.^22^, ^23^, ^24^, ^25^, ^26^, ^27^^,^^29^, ^30^, ^31^^,^^37^^,^^44^ A systemic whole-of-government approach is needed to address inadequate investment in mental illness more broadly and particularly for illness groups such as EDs where complex structural limitations of disease burden and mortality reporting exist. Affirmative action will involve the building of service capacity (which will grow research capacity), the establishment of dedicated funding initiatives (rather than redistribution of existing mental health funds), and a focus on funding novel therapeutics. For EDs, rapid standardisation of data collection practices (including outcome measures) across healthcare services, inclusion in national health surveys and the establishment of a Clinical Quality Registry are urgently needed to overcome existing epistemic barriers to investment. Allocation of research funding should be considered on an empirical basis and commensurate with indices such as mortality and disability burden only where those indices are accurate. This will ensure investments made in mental health reflect overall need and are informed by the best available evidence.

## Contributors

EB: conceptualization, methodology, formal analysis, investigation, writing—original draft, visualization, and project administration. NK: formal analysis, writing—original draft, review and editing. JM: investigation and writing—original draft, review and editing. PM: investigation, writing—review and editing. IH: writing—review and editing. SM: conceptualization, methodology, investigation, writing—review and editing. All authors contributed to the article and approved the submitted version.

## Data sharing statement

The datasets used and/or analysed during the current study are available from the corresponding author on reasonable request.

## Declaration of interests

All work for this study was supported by the Australian Government Department of Health and the Translation of Eating Disorder Evidence into Clinical Practice 4-G5CD4SY grant. IH is supported by NHMRC Centre for Research Excellence grants (APP1171910 and APP1061043) and an Australian Government funded Project Synergy grant. He is on the Janssen Cilag advisory board and presents and chairs their online webinars and educational events; the MRFF Australian Medical Research Advisory Board; an unpaid member of the board of Psychosis Australia Trust; Member of the Clinical Advisory Group for the evaluation of the Better Access to Psychiatrists, Psychologists and GeneralPractitioners through the Medicare Benefits Schedule (MBS) initiative (Dept of Health). IH is Chief Scientific Advisor and an equity shareholder in Innowell PTY LTD. NK is supported by a University of Sydney Faculty of Medicine and Health Executive Dean Stipend Scholarship and has received travel funding support from the University of Sydney and the Nutrition Society of Australia. He is on the advisory and awards committees for the American Academy of Nutrition and Dietetics Council on Research.
